# Food from Earth: Sustainable Farming in Action

**DOI:** 10.1289/ehp.121-a212

**Published:** 2013-07-01

**Authors:** Wendee Nicole

**Affiliations:** **Wendee Nicole**, based in Houston, TX, won this year’s American Society of Journalists and Authors award for best science magazine article.

With a lurch, the motorized banana tram starts moving slowly along its simple track carrying 100 banana bunches—cornucopias of green fruits, cloaked in blue plastic bags to protect them from insects and sun damage. A Costa Rican worker in a hard hat sits at the helm guiding the tram—nicknamed “the spider”—toward the packing plant a couple miles away. The tram driver and other workers harvested these bananas with machetes on the 813-acre banana plantation at EARTH University (Escuela de Agricultura de la Región Tropical Húmeda) in the Caribbean lowlands of Costa Rica.^^1^^

**Figure:**
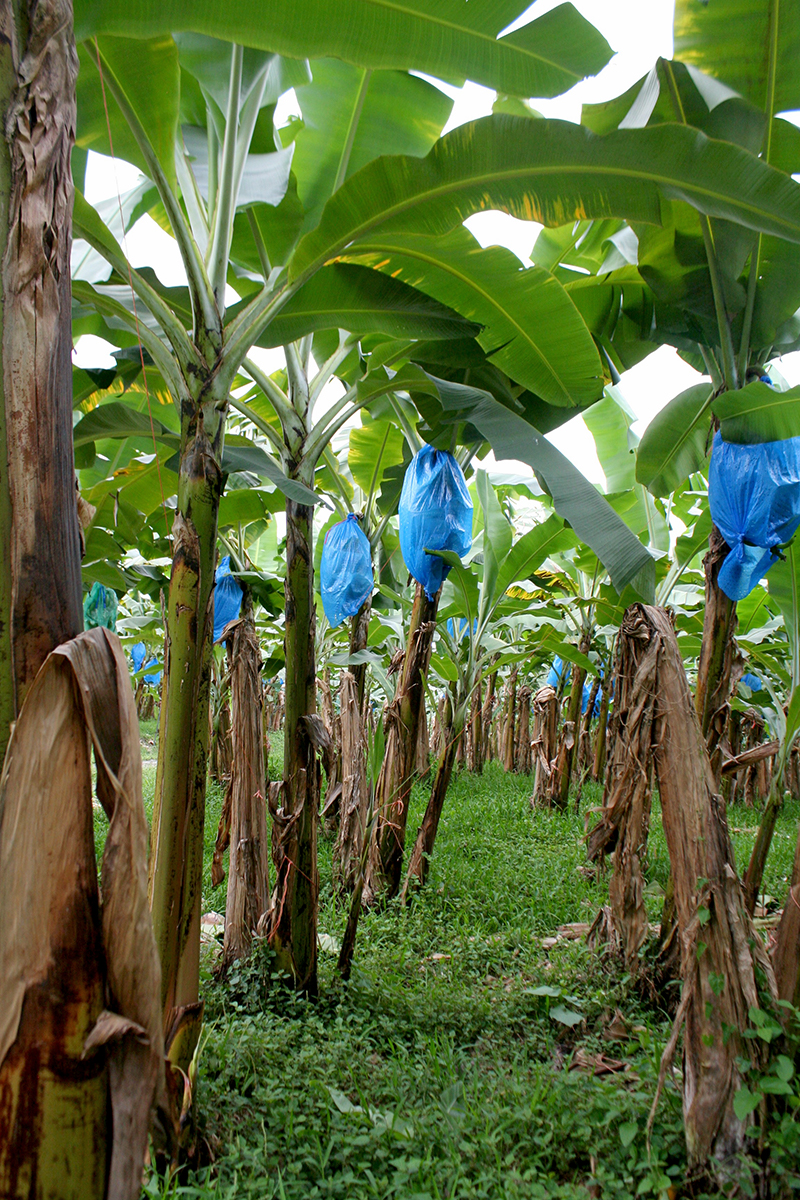
The banana is the world’s largest herbaceous flowering plant. Commercial bananas are sterile and are propagated by planting root stock from donor plants (the dark specks in the center of a banana are undeveloped ovules, the precursors to seeds). © 2013 Wendee Nicole

With its row upon row of genetically identical Cavendish banana plants stretching for acres, one might mistake EARTH’s plantation for a traditional banana farm. But through trial, error, and scientific research, EARTH University—funded largely by a U.S. Agency for International Development (USAID) endowment—has created a more ecologically friendly, lower-pesticide (but not organic) banana since the university was established in 1989. The university is also impacting the larger banana industry and nations around the world as alumni take sustainable agriculture initiatives and new business opportunities back to their home countries.

## Education, Not Violence

Costa Rica is the world’s second largest exporter of bananas,^^2^^ and the banana trade contributes heavily to the nation’s gross domestic product,^^3^^ making EARTH’s activities part of an economically important industry in Latin America. EARTH bananas are now stocked in 85% of Whole Foods Market grocery stores in the United States and are sold in European and local markets as well.

However, banana farming is just one facet of the university, whose idealistic mission—to prepare leaders “to contribute to the sustainable development of the tropics and to construct a prosperous and just society”^^4^^—originated as a dream to lift rural youth throughout the region out of poverty during the political turmoil that plagued Latin America in the 1980s.^^5^^

“[At that time] there was a lot of political insecurity in Central America, and the U.S. government was trying to stop the advance of communism. They wanted to support Costa Rica, which was being influenced by what was happening,” explains José Zaglul, who has served as the president of EARTH University since the beginning. USAID was already providing money to stabilize the democratic enclave. (Costa Rica had abolished its military in 1949, instead channeling money into education.^^6^^) Monge declared neutrality in the region’s Contra–Sandinista battles in 1983.^^5^^^^,^^^^7^^

Amidst the geopolitical strife, Costa Rican businessman Rodolfo Cortés had a vision to create a school that would help protect the rapidly disappearing rainforest by finding sustainable ways to cultivate crops without devastating natural resources. The school would also educate future leaders who would promote peace and democracy.

**Figure:**
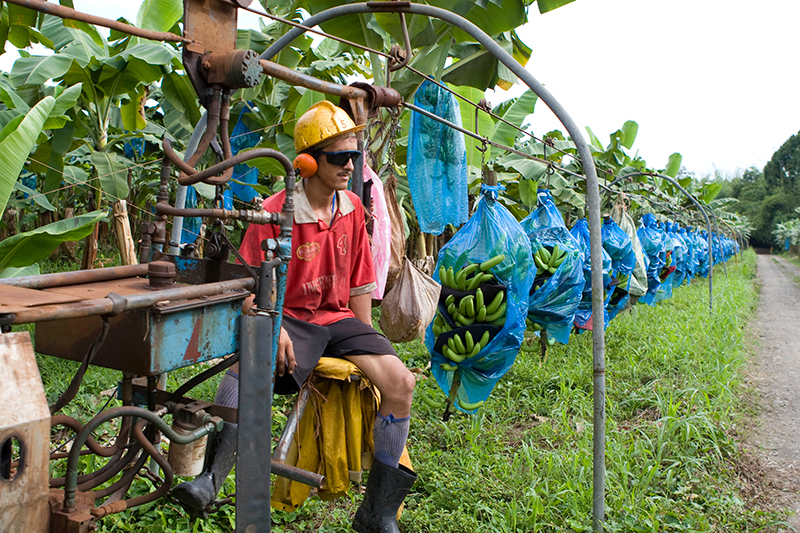
A tram driver ferries banana bunches from the field to the packing plant on a mechanized train. On traditional farms, laborers must use pulley systems to drag up to 25 bunches at a time for more than a mile in extreme heat, humidity, and rain. © 2013 Angela P. Johnson

“[Violence] happens because of poverty and the desperation of people,” Zaglul says. “[Costa Rica] didn’t want to receive money for military aid, and [the EARTH founders] said, ‘Why don’t we create an academic institution?’ Emphasizing education is what Costa Rica has done traditionally.”

Cortés shared his vision with a handful of others, who brought the idea to Luis Alberto Monge, then the president of Costa Rica. The concept received Monge’s blessing, and after completion of a feasibility study, USAID provided funds for land, equipment, campus construction, and—for the first time in the agency’s history—a university endowment that would sustain EARTH over the long term. But the school concept proved controversial among Costa Ricans; many questioned U.S. motives, and the nation’s Congress was unable to pass authorizing legislation. “They saw it as a political move by the U.S. to support the Contras,” Zaglul says. Finally, in 1986 Monge’s successor, Oscar Arias Sánchez—who would receive the 1987 Nobel Peace prize for his efforts in stabilizing the war-torn region—passed legislation establishing EARTH as a private, nonprofit university.

**Figure:**
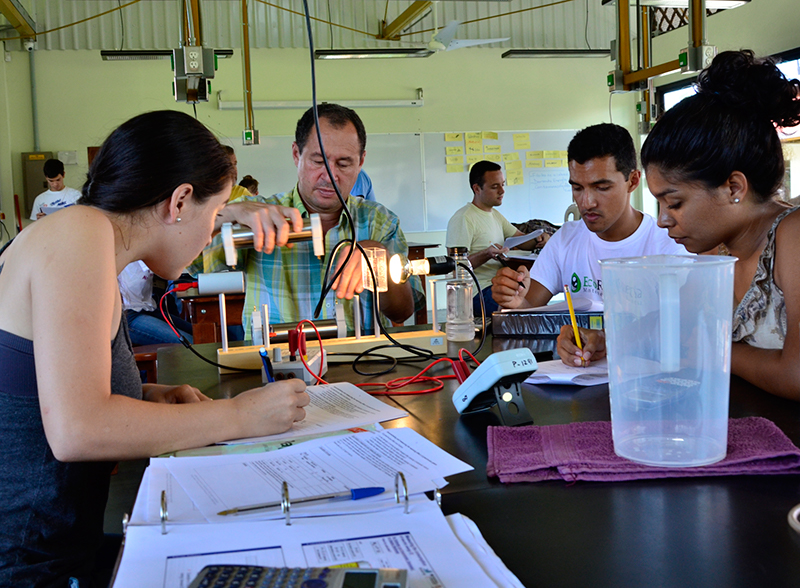
Students in this course are studying renewable energy systems for rural areas. EARTH University is home to the only renewable energy laboratory in Central America and offers training seminars for professionals in the region as well as its students. © EARTH University

**Figure:**
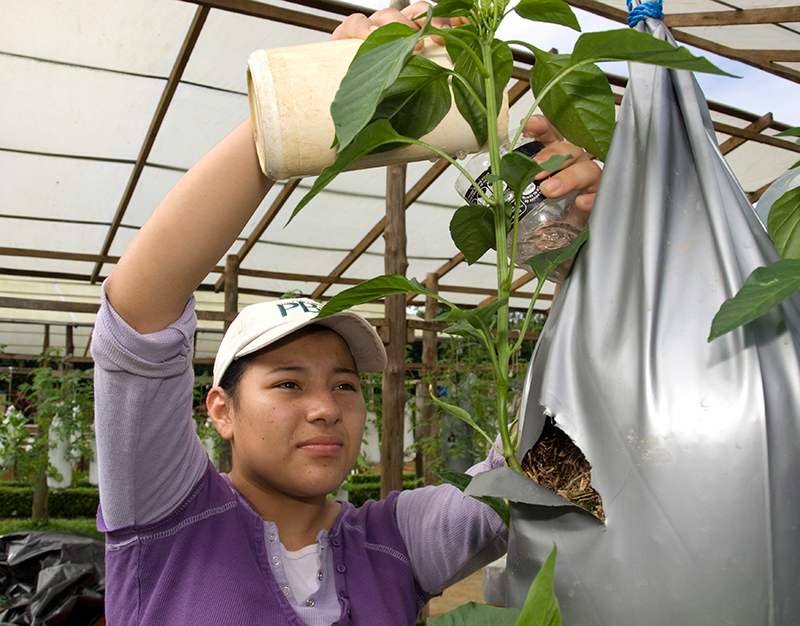
A student applies nutrients to hanging crops growing in EARTH’s peri-urban agriculture module. This system provides a way to grow nonnative vegetables that are commonly used in the local diet, such as tomatoes, bell peppers, celery, onions, and cilantro. © EARTH University

**Figure:**
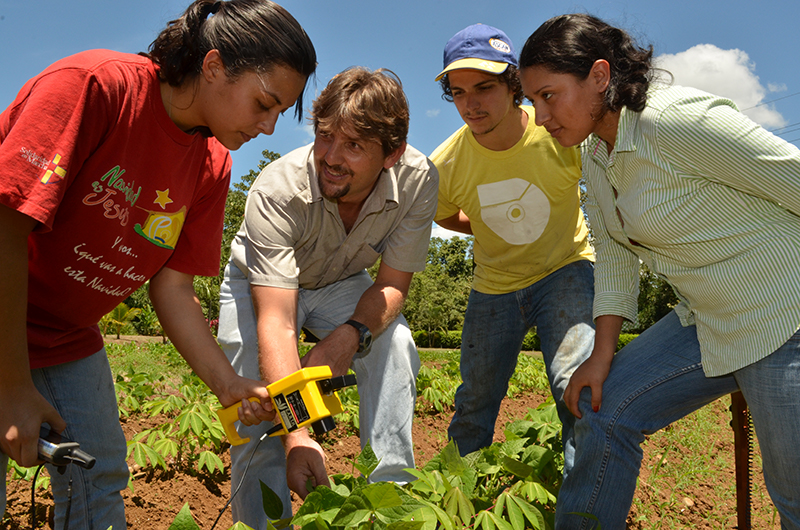
Students in a soils course explore the concept of precision agriculture, which emphasizes observation of variability within individual farm fields to optimize crop management. Here the students evaluate the health of a cassava crop using a chlorophyll meter, which indirectly measures the amount of nitrogen in the plant. © EARTH University

By April 1989 EARTH’s founders had hired Zaglul as president, and after a whirlwind recruiting trip through Latin America and rapid construction of the campus on a former cattle and banana farm near the town of Guácimo, classes began in March 1990 with 60 students. Despite the initial resistance, time has shown the political neutrality of the university and the sincerity of its goals, Zaglul says.

## “Upside Down” Education

“We’re trying to find students who would otherwise never be able to go to college,” says Kristine Jiménez, EARTH’s director of communications. Professors and faculty travel to each country to talk to prospective students in person, encourage their application, and later return to interview promising applicants. Students are selected based on their commitment to environmental sustainability and social justice, personal values, and leadership potential.

“The most effective way of alleviating poverty is to give opportunities to kids who come from poor areas,” says Zaglul. “Education is the greatest tool we have.”

But recruiting primarily rural, poor students meant the university had to educate differently from the outset. At first, Zaglul says, “My board was very concerned. How do you bring [students] up to university level when they come not only from disadvantaged families but also disadvantaged high schools? It’s not that they aren’t intelligent, they just have not had the opportunity to be well educated.” So EARTH created an “upside down” curriculum that, from the start, puts students in the field and on the farm to provide a context for the more theoretical aspects of agricultural science that they learn later in classroom studies.

In their first year, students work on a sustainable vegetable garden that produces food for the cafeteria, spend time on the campus’s dairy and pig farm, and work with waste management, including recycling and learning how to operate biodigesters that harvest biogas from human and animal waste on the campus. When needed, students receive remedial math and computer training.

**Figure:**
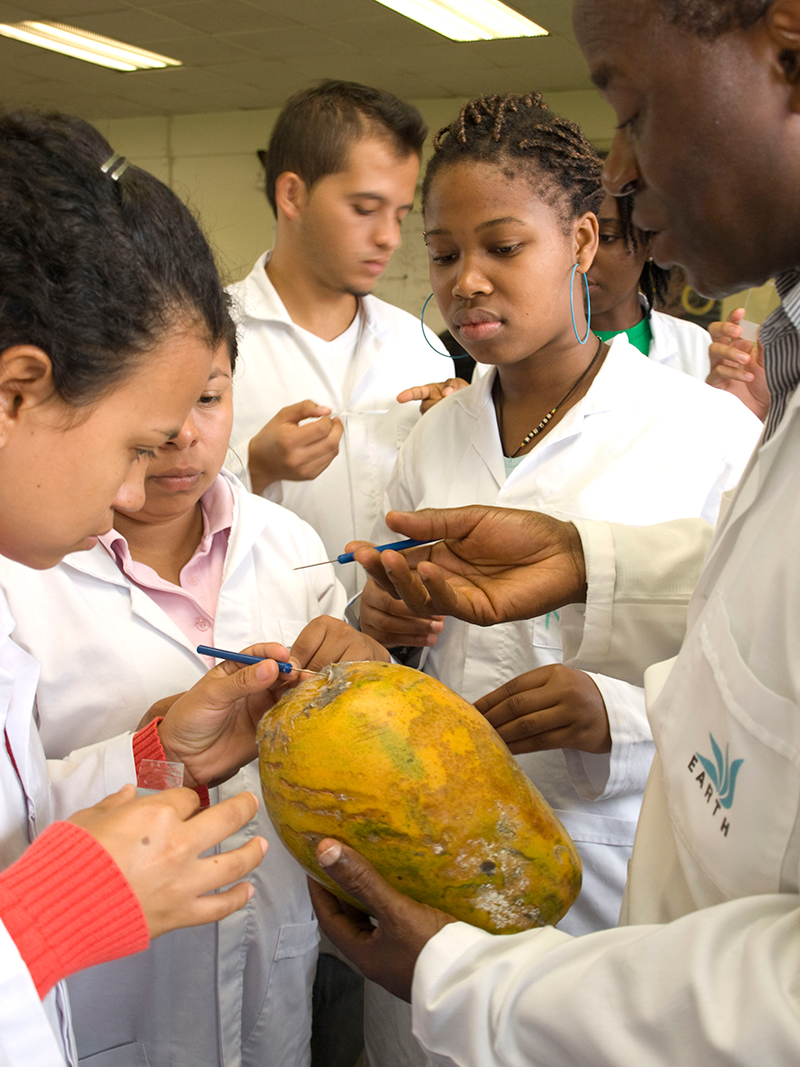
Students in a course on disease management take samples of papaya skin as part of a laboratory exercise. © EARTH University

**Figure:**
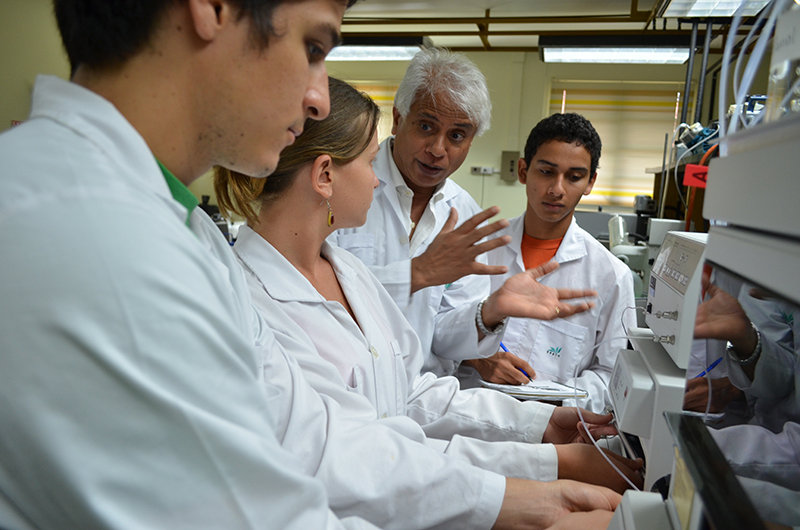
An EARTH professor guides students doing analysis in the university’s Soils Laboratory. © EARTH University

By the end of their first year, student groups of four to six students each receive up to $5,000 to develop and run a company. They create a business plan, conduct a feasibility study, and then run their companies for a year. Successful projects have included yogurt derived from the campus dairy and chocolate-covered pineapples and bananas (all still sold on the campus) as well as an agro-tourism company called Karibu, which held community workshops and took foreign and local visitors on tours of the campus ethnobotanic and peri-urban gardens.

“When a project closes, assuming they have profits, they first have to pay their labor and all their expenses, and they have to pay an environmental fee if they’ve used any kind of agrochemical, since EARTH is incentivizing sustainability,” says Jiménez. At the end, after they pay back the loan, the group keeps two-thirds of their net profits, and a third goes into a revolving fund that covers insolvent projects. Karibu—the most profitable student project to date—not only paid off its original loan with interest but also netted $1,300 in profit for each of six group members.

In the third year, students participate in an internship, often in their home country, and spend seven weeks at EARTH’s La Flor campus in the dry tropical province of Guanacaste on the opposite side of the country. There they live with host families, work in local businesses, and engage in community service. By their final year, students return to the Guácimo campus for a full load of advanced classes.

Around 100 students enter EARTH each year, and approximately 83% graduate four or five years later with a *licenciatura*, which is between a bachelor’s and a master’s degree in agricultural sciences. “We have proven that depending on SAT exams is not the greatest thing in the world,” says Zaglul.

**Figure:**
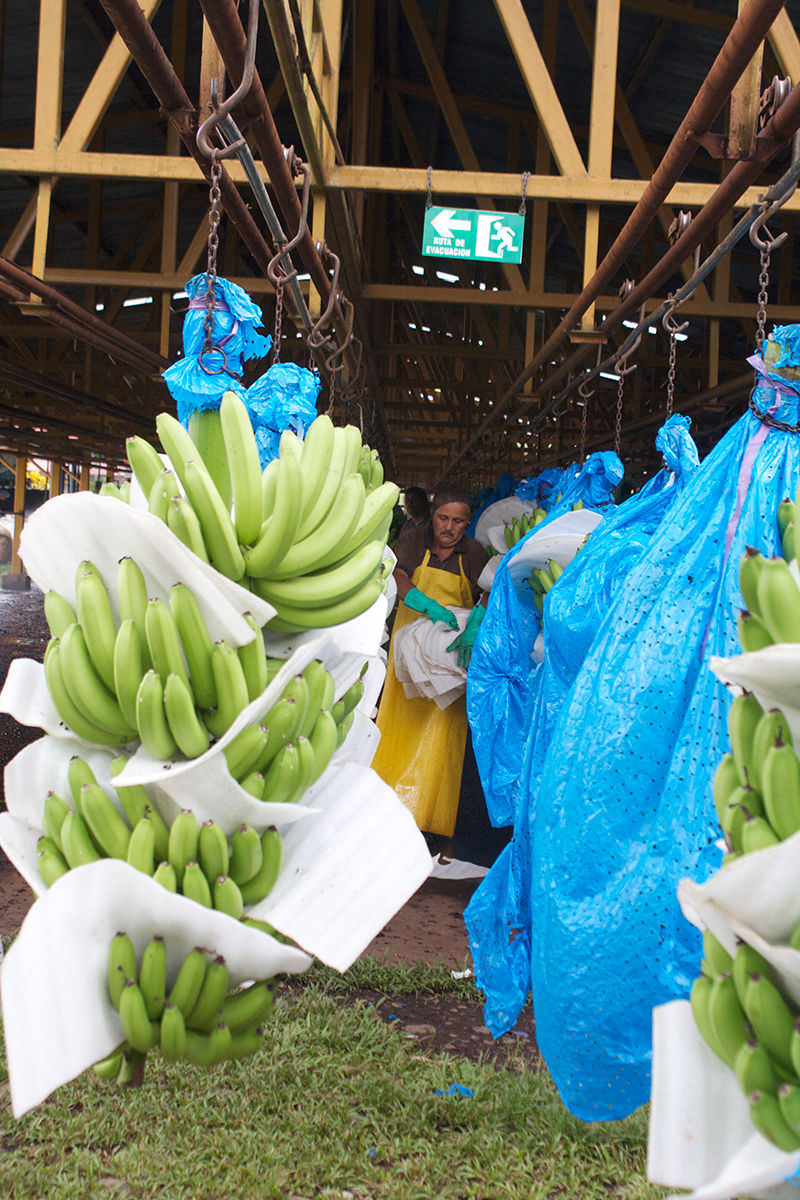
Padding is placed between the layers of bananas to prevent bruising during the fruit’s growth, harvesting, and transportation to the packing plant. © 2013 Wendee Nicole

**Figure:**
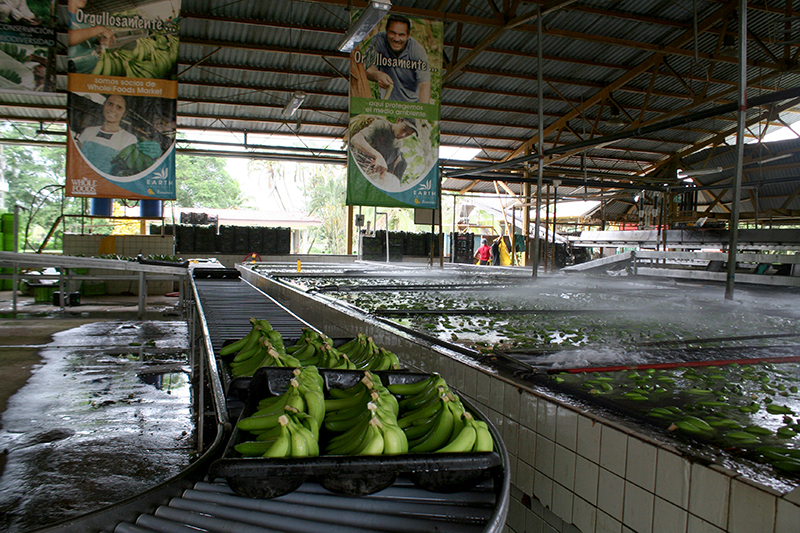
After harvest, individual “hands” of bananas are cut off the bunch, then bathed in water and treated to repel fungi. © 2013 Wendee Nicole

Until 2012 more than 90% of EARTH University students hailed from Latin America and the Caribbean, but the university has recently begun outreach to Africa, and for the 2012 and 2013 entering classes, Africans represent 17% of the student body. Sixty percent of EARTH’s students—many of whom could not afford a university education—receive full scholarships, including room and board, while another 30–40% receive partial scholarships.

## An Alternative to Conventional Banana Farming

When EARTH bought the land near Guácimo, the banana industry had a reputation for causing harm to human and environmental health due primarily to heavy pesticide use, which had resulted in lawsuits and negative publicity.^^8^^^^,^^^^9^^^^,^^^^10^^^^,^^^^11^^ Plantations also regularly discarded banana harvest trash on the ground, including the protective plastic bags and the cords used to keep the shallow-rooted banana plants upright. During torrential downpours, this trash would wash into rivers and out to sea.^^12^^ “The river near the campus was totally blue with plastic when [we] started,” Zaglul said in a 2010 keynote address before the Association for the Advancement of Sustainability in Higher Education.^^13^^

Prior to 1960, plantations around the world grew the ‘Gros Michel’ banana cultivar. But the spread of fungicide-resistant Panama disease, which attacks the roots and kills the plants, wiped out the Gros Michel, devastating the entire banana industry.^^14^^ Banana farmers rebounded with the adoption of Cavendish cultivars, which are immune to Panama disease.^^15^^

But Cavendish bananas, it turned out, are vulnerable to a different fungus, black Sigatoka, which attacks the leaves. Without pesticide treatment, black Sigatoka leads to smaller, lower-quality banana bunches.^^16^^^^,^^^^17^^ To manage the fungus, traditional plantations apply up to 40 kg of chemicals per hectare per year—10 times the typical chemical load used on intensive agriculture of other crops in developed nations.^^17^^^^,^^^^18^^^^,^^^^19^^ The chemicals used vary by country but have included the nematicides 1,2-dibromo-3-chloropropane, terbufos, fenamiphos, cadusafos, carbofuran, and ethoprophos; the fungicides thiabendazole, propiconazole, and imazalil; the insecticide chlorpyrifos; and the herbicide paraquat.

Conventional banana farms use protective plastic bags that have been impregnated with insecticides (typically chlorpyrifos), apply nematicides and fungicides via cropduster or by injection into the soil, and use herbicides to kill competing plants. As Zaglul said in his 2010 address, “They used to spray the banana plantations with chemicals by plane—and the workers stood in the fields with flags showing the pilot where to spray.”^^13^^ Research has linked chemicals used on conventional banana farms to acute poisoning and death,^^20^^^^,^^^^21^^^^,^^^^22^^ sterility,^^23^^ and cancer^^24^^^^,^^^^25^^^^,^^^^26^^^^,^^^^27^^ among agricultural workers. In U.S. studies of urban pesticide use, chlorpyrifos exposure has been associated with neurological impairment in children.^^28^^^^,^^^^29^^

**Figure:**
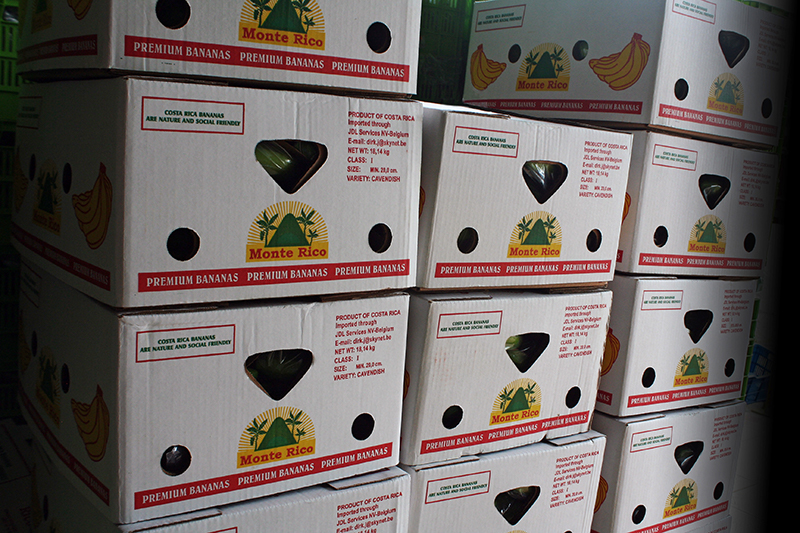
The finished bananas are packed in boxes and shipped to markets locally and abroad. EARTH bananas are stocked in most Whole Foods Market grocery stores in the United States. © 2013 Wendee Nicole

**Figure:**
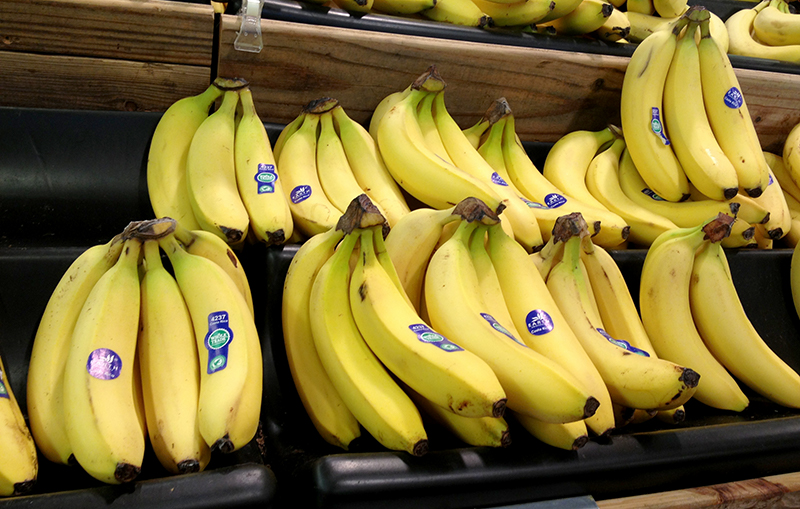
Author Wendee Nicole spotted these EARTH bananas at a Whole Foods Market in Houston. © 2013 Wendee Nicole

So what would a school dedicated to sustainability do with a banana farm? “We were advised to get rid of it,” says Michelle Medina, who creates new business relationships for Variedades del Trópico Húmedo, the university’s for-profit arm (profits are funneled into EARTH scholarships). In an act that helped define EARTH’s commitment to transforming agriculture in practical ways, the university ignored the consultant’s advice to ditch the farm. “President [Zaglul] decided that we’re a sustainable school, and if we’re going to teach students that they can produce sustainably, we need to enter the market with the exact same pressures as any other company,” says Medina.

Over the past three decades, EARTH University’s professors and students have researched how to improve environmental sustainability and worker safety on the farm.^^30^^ Everything from reducing chemical use to recycling the plastic bags to making onsite banana production carbon neutral has been studied, tested, and eventually implemented when it made business sense.

## Shift to Sustainability

The banana-tram crosses the main road entering the university, often stopping traffic for “banana crossings.” To improve worker conditions, EARTH installed more than 30 miles of track several years ago. On traditional farms without motorized trams, laborers use pulley systems to drag up to 25 bunches at a time for more than a mile in extreme heat, humidity, and rain; at 60 pounds per bunch, this is grueling work.

Medina gestures toward the banana farm, with row upon row of stately banana plants. Birds fly overhead, and birdsong arises from the nearby second-growth forest, adjacent to the Dos Novillos River. “You can see here we have river, forest, forest, forest, and plantation,” she says. “We do not plant within fifty feet of the river.” The riparian buffer protects the stream from chemical runoff and erosion, and allows for more wildlife habitat. Monkeys, toucans, agouti, sloths, and wild cats live here, unlike on traditional plantations, which stretch for miles, unbroken monocultures inhospitable to wildlife.^^31^^ EARTH’s farm contains multiple blocks of banana plants interspersed with regrowing forest, which has earned the university’s bananas certification by the Rainforest Alliance.

When the farm began operating under the university’s guidance in 1991, it started recycling its plastic bags and cords. The university asked other banana companies to recycle as well,^^32^^ pointing out the external costs of waste to the communities, but met steep resistance, according to Zaglul. Since then, however, plastic bag reuse and recycling have become industry-standard.^^33^^^^,^^^^34^^^^,^^^^35^^

Piles of banana stalks left after harvest create another source of copious waste, and in 1991, EARTH began recycling the stalks into banana paper, sold in the campus gift shop as journals and stationery. Any leftover banana waste is composted, and the compost eventually goes back to the banana farm or the vegetable gardens.

Over the years, EARTH has also systematically researched how to reduce and optimize chemical use on its plantation. Because fungi attack voraciously in the humid tropics, EARTH still aerially sprays the fungicides mancozeb and tridemorph once or twice a week for three weeks of the month, but on the final week they spray a proprietary blend of bacteria and yeast species (so-called effective microorganisms). This has cut the university’s chemical use by more than a quarter.

Crop dusters hired by EARTH use Global Positioning System devices to ensure the chemicals target the precise location of the banana plots. “We spray when there’s no one in the field,” says Medina. “We send out e-mails to every single person on campus … and we also have warning signs in all the fields to keep people out during spraying.” People must stay out of the fields for at least two hours afterward.

Starting in 2005 EARTH replaced treated plastic bags with chili pepper and garlic to repel insects. And in 2006 the university stopped using herbicides in the fields; workers now manually remove weeds and plant nitrogen-fixing vegetation in between rows to improve soil health and prevent erosion. (Jiménez had no data on how these strategies have worked so far.)

A mold called “crown rot” often grows on the stems of post-harvest bananas, and most packing plants use a chemical fungicide to ward it off. In 2006 EARTH replaced some uses of the fungicide imazalil with Biocto 6, an organic fungus repellant. Biocto 6 contains primarily citrus seed extract and a wax-based additive called Verdiol.^^36^^ (Jiménez says European law still requires that imazalil be used on bananas sold there.) When the tram driver reaches the packing plant, workers begin removing the plastic bags and foam inserts placed inside each bunch to protect individual “hands” (clusters of bananas) from damage. After the banana hands are cut off the bunch, they are bathed in water, brushed with Biocto 6, and packed into boxes.

**Figure:**
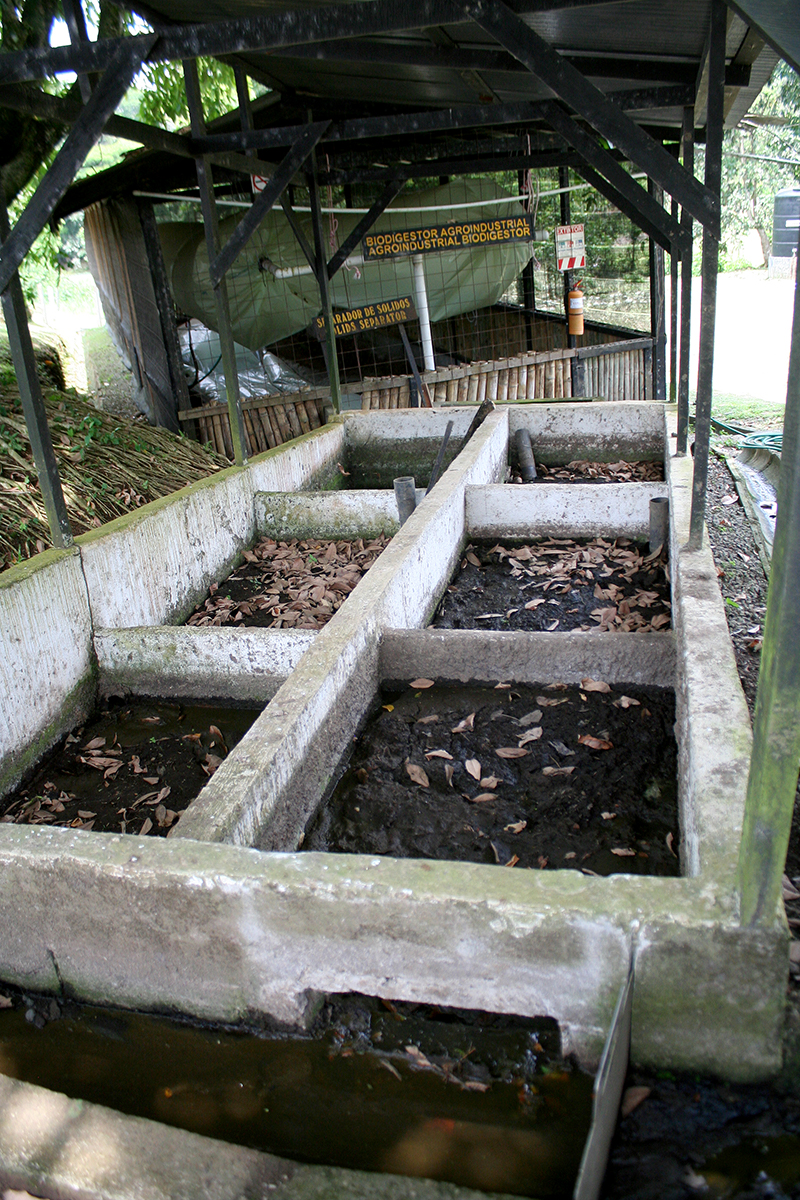
Six biodigesters on the EARTH campus convert animal and human waste to fertilizer and biogas. Over the past few years, EARTH has also installed more than 2,000 biodigesters for farms and businesses around Costa Rica. © 2013 Wendee Nicole

EARTH University has shown it is possible to make banana production more sustainable, provide better working conditions, and still make bank—the banana farm and a handful of other EARTH products net around US$1 million per year. The farm, which is run largely by nonstudent workers, pays its employees a living wage—a condition for Rainforest Alliance certification, although EARTH instituted fair labor practices from the outset, independent of seeking certification.

EARTH was only breaking even before it partnered with Whole Foods Market, says Matt Rogers, associate global produce coordinator for the grocery chain, but it wasn’t because they didn’t have a strong product to sell or that they didn’t know how to run a profitable business. “They didn’t want to sell their bananas to multinational banana brands that would have requested changes to the way they produced their bananas,” he explains. EARTH stood firm, and it paid off. Their bananas became Whole Foods’ first Whole Trade® product, guaranteeing customers fair worker conditions and environmental sustainability.

For the past several years and funded in part by Whole Foods Market, Inc., EARTH has operated two 10-acre research plots on which faculty have grown organic bananas, a designation requiring that no synthetic pesticides be used. So far, the fruits remain too small for export quality, but research continues. The university is also researching the integration of other sources of income, such as cacao.

## Ripple Effect

EARTH’s research and outreach activities have influenced not only the surrounding community, but also the greater agriculture industry in many different areas, from working toward carbon neutrality to creating new sustainable products, many of them pioneered by students. One international success story is EARTH’s coffee, co-branded with Allegro, which is now sold in Whole Foods stores. EARTH alumna Hortensia Solís got a job as sustainability manager for the Coffee Cooperative of Dota (Costa Rica) and, beginning in 2009, helped the co-op develop a carbon-reduction strategy that in 2011 earned its beans certification by Carbon Clear.^^37^^

A recent initiative making waves is the Carbon Neutral project, led by EARTH professor Edmundo Castro. EARTH systematically reviewed each of the university’s activities and calculated its carbon footprint and in 2007 achieved carbon neutrality. Currently, the campus emits 1,704 metric tons of carbon dioxide equivalent and sequesters 26,182 metric tons annually, offsetting not only the Guácimo campus and banana farm but also the La Flor campus and the EARTH University Foundation in Atlanta, Georgia. The university sells the remaining carbon credits to Costa Rican businesses that wish to achieve carbon neutrality.

EARTH’s efforts tie in to the Costa Rican government’s initiative to become the world’s first carbon-neutral nation by 2021.^^38^^ Although some have criticized carbon offsets as merely assuaging guilt and allowing energy waste to continue,^^39^^^^,^^^^40^^ EARTH takes seriously its commitment to reduce emissions, both within its own campuses and in working with other businesses, according to Castro, who says, “There is an internal responsibility to reduce emissions.”

The campus itself reuses or recycles 83% of the waste it generates. Human and animal waste is diverted to small-scale biodigesters that both treat wastewater and produce biogas. There are many types of biodigesters; EARTH uses a simple polyethylene bag, says professor Rebeca García, a specialist in municipal waste. “There’s an initial system that separates hard solids—manure, fiber, and such—which is kept in sedimentation tanks, and that waste is used to create ‘worm compost,’” she explains. The liquid enters the long plastic bags, where anaerobic bacteria break down organic matter and release methane and carbon dioxide. This biogas fuels the cafeteria and provides some electricity for the dairy farm.

Depending on the organic matter fed into the biodigester, the nutrient-rich liquid remaining after gas production may be used on crops. “The use on crops, however, should be controlled because of the nitrogen and phosphorous content, which could on large farms contaminate underground water supplies,” Jiménez says.

EARTH has six biodigesters around campus. However, the university’s outreach has an even bigger impact: Over the past few years, EARTH has installed more than 2,000 biodigesters for farms and businesses around Costa Rica.

Stephen Brooks worked with EARTH to install a manure biodigester more than 10 years ago at the Punta Mona Center for Sustainable Living and Education, which he founded. “The professor came with his whole class to Punta Mona to install the biodigester, and we exchanged with them educational information on permaculture,” says Brooks. A couple years ago, he consulted with EARTH to install “Central America’s largest privately-owned biodigester” at the new La Ecovilla sustainable development community near San Mateo de Orotina, Costa Rica.^^41^^

And EARTH’s reach may expand further. “Right now we are starting to develop prototypes for biodigesters that use agricultural refuse from banana, pineapple, and coffee production,” says Bert Kohlmann, a professor at EARTH and director of its Center for Research and Development of Renewable Energies. “From the very first results, vegetable matter produces more biogas than animal manure, and it seems it produces better-quality biogas.” Kohlmann and colleagues are seeking funding to build a prototype.

## Future of EARTH

Although EARTH is a small enclave in a tiny country, Castro, who works alongside Kohlmann in their sustainability initiatives, believes in the power of example. “If you can give an example to your neighbors and show them that you can produce also by reducing pesticides, that is going to be important for the consumers,” he says.

That’s not the only example of leadership, Kohlmann says. “Twenty years ago, if you came to the rivers in this part of Costa Rica, you are going to find a lot of plastics in the rivers. And now you see that the rivers are very clean because EARTH University started with the first students cleaning and picking all the plastics from the rivers,” he says. Although littering of the bags is now illegal, bags from other large plantations do still end up in rivers and oceans, according to Kohlmann. Nevertheless, he says, “now the other banana producers, they don’t throw the plastics in the rivers as [much as] they used to.”

Although professors guide the research, development of business products, community outreach, and sustainability initiatives, with a 10-to-1 student–faculty ratio, the students play an integral role in all these endeavors. “Students come here knowing they want to go back to their countries and make a positive difference, and so everything they’re learning, they take it with such seriousness,” says Jiménez. “It’s very unusual to find a place where everyone is so focused and dedicated to a mission. It’s very powerful for the people who work here, and for the students.”

The original vision of the university founders involved lifting people out of poverty through education, not just so they could get jobs after graduation but so they could create opportunities for others. And based on the success stories of many alumni—88% of whom work in their home countries—it would seem the vision is bearing fruit.^^42^^
